# COVID-19 Reinfection

**DOI:** 10.7759/cureus.12730

**Published:** 2021-01-15

**Authors:** Mohammed I Habadi, Tarig H Balla Abdalla, Nashaat Hamza, Afnan Al-Gedeei

**Affiliations:** 1 Family Medicine, International Medical Center, Jeddah, SAU; 2 Family Medicine, University of Jeddah, Jeddah, SAU; 3 Infectious Disease, International Cancer Center, Jeddah, SAU

**Keywords:** covid 19, covid-19 reinfection, reinfection rate, ksa covid 19, covid-19 reinfection in ksa

## Abstract

The possibilities of coronavirus disease 2019 (COVID-19) to reinfect individuals have not been reported yet. All reported hypothesis of reinfection has been attributed to either relapse of the disease or having a mild course of symptoms. We report two cases of COVID-19 positive patients where they had complete resolution of symptoms and negative COVID-19 results. Weeks later, they returned with milder symptoms and a positive COVID-19 culture swab. In conclusion, early stages of COVID-19 where mild signs and symptoms are reported can be prolonged and the virus can stay dormant in the body for relapse later on.

## Introduction

Ever since the emergence of the severe acute respiratory syndrome coronavirus-2 (SARS-CoV-2) commonly known as coronavirus disease 2019 (COVID-19), the world has been on high alert whether limiting travel or implementing lockdowns to decrease the speed of spread of the virus in the community. As time passes, discovering the likelihood of reinfection can greatly affect how we approach the vaccine race and if the herd immunity is actually achievable [1]. Moreover, there has been around 80 genetic reported variations of the virus, which makes the idea of an effective vaccine being far from reach scientifically [2]. Reinfection possibility has been long feared and was denied by many papers, either in animal or human subjects; as such an event would prove that vaccine is a milestone that may never be reached [3]. However, to this day, all reported possibilities of reinfection were reported as cases of either under-detecting or the assumption of self-resolution of the disease after home quarantine [4]. Other studies also reported that many clinically confirmed cases can reach up to four negative sputum cultures as another cause of assuming the reinfection status of COVID-19 [4]. Lastly, there have been many documented cases of persistent COVID-19 symptoms including cough, fever, and shortness of breath with a reported mean of 60 days [5].

## Case presentation

First case

**Figure 1:**
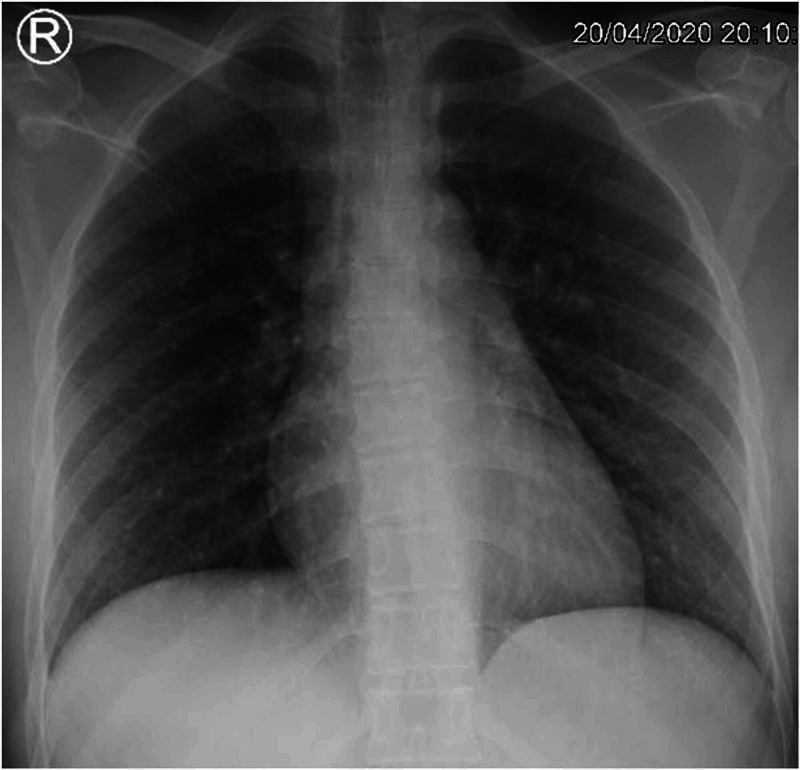
Chest X-ray upon admission for case 1.

A 44-year-old-female healthcare worker presented on April 20, 2020 to the International Medical Center in Jeddah, Saudi Arabia to the ED complaining of fever with chills, severe sore throat, fatigue, and change in appetite for two days. She denies any history of cough, sputum production, shortness of breath, loss of smell, abdominal pain, nausea, vomiting, or diarrhea for the past 48 hours prior to the ED visit. Upon examination, she was slightly febrile with a temperature of 37.8 degree celsius and an increased heart rate of 121 beats/minute. Throat examination showed congested throat and nasal mucosa with clear chest examination while systemically other physical examinations were unremarkable. Regarding her blood work, it included a complete blood count (CBC) and chest X-ray (CXR) which showed no abnormalities (Figure 1). Furthermore, a COVID-19 nasopharyngeal swab was taken and sent to the same facility laboratory which revealed a positive detection of the COVID-19 virus. As a result, she was admitted as a confirmed case of COVID-19/acute nasopharyngitis.

During her hospital course, the patient started complaining of left ear pain, which was diagnosed with otitis media, and abdominal discomfort with no bowel habits alteration with no other noted events. The patient received supportive treatment of analgesics for ear pain and initially treated for otitis media with amoxicillin-clavulanate (1000 mg/200 mg), which was shifted to levofloxacin (750 mg) on her third day of admission. Another swab was taken on her fifth day during admission which was negative for COVID-19. Another swab was taken 14 days after discharge from our hospital to home isolation which returned back negative. Four months later, the patient returned back to the ED complaining of runny nose and severe persistent productive cough and loss of smell and partial loss of taste. The patient was completely asymptomatic during the four months prior to this presentation. The patient denied any shortness of breath, sore throat, or fever. On examination, her vital signs were normal. Throat examination showed congested throat and nasal mucosa with clear chest examination while systemically, other physical examinations were unremarkable. Naso-pharyngeal swabs were taken for influenza A, influenza B, and COVID-19, and sent to the same facility laboratory which came back negative except for COVID-19 (Table 1). So she was diagnosed as a case of COVID-19/acute nasopharyngitis reinfection. The patient was treated symptomatically with paracetamol, vitamin C tablets, and zinc supplements, and she was given pulse oximetry for monitoring her oxygen saturation and advised for home isolation for 14 days.

**Table 1:** Sequence of the first case COVID-19 test results.

Dates	20/04/2020	25/04/2020	14/05/2020	06/08/2020
COVID-19 test result	Positive	Negative	Negative	Positive

**Figure 2:**
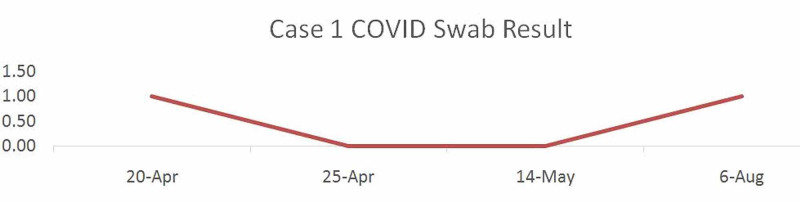
Sequence of the first case COVID-19 test results.

After compliance to quarantine guidelines, the patient presented to the hospital’s outpatient clinic after home isolation of 14 days for reassessment which reported a clear CXR and improvement of the symptoms (Figure 2).

**Second case**

On April 19, 2020, a 35-year-old, heavy smoker, with no known significant past medical history presented to the emergency department reporting unprotected contact with a COVID-19 case. He denied any history of fever, fatigue, cough, shortness of breath, nausea, vomiting, or diarrhea. His physical examination and vital signs were normal. Due to the unprotected contact with a COVID-19 confirmed case, a nasopharyngeal swab was taken for this patient and he was advised for home isolation. Three days later, the test result confirmed COVID-19 infection, and was admitted for further investigations. During his hospital course, the patient did not report any new or worsening symptoms and his physical examination was unremarkable. His blood tests revealed a high lymphocyte count but low neutrophil count (Table 2). After two days of admission, the patient was discharged to an isolation facility.

**Table 2:** Blood test results for case 2. CBC, complete blood count; WBC, white blood cell; HS-CRP, high-sensitivity C-reactive protein

Investigation	Result	Reference range
CBC	-WBC 5.76 Neutrophils percentage (39.7%) - Lymphocytes percentage (50.7%)	WBC 4-11 *10^9^/l Neutrophil 40%-73%. Lymphocytes 18%-45%
HS-CRP	5.12 mg/L	0-5 mg/L
Serum Procalcitonin	0.06 ng/mL	0.5-2 ng/mL
D-Dimer	0.32 mg/L	0-0.55 mg/L

On May 9, 2020, the patient presented to the hospital for reassessment for returning back to work where another swab was taken and was negative. One month later, on the 12th of July 2020, the patient complained of fever, cough, body ache, abdominal pain, and loss of taste for eight days duration with no shortness of breath, vomiting, or diarrhea. Upon physical examination, he was febrile with a temperature of 38 degree celsius with no other abnormalities. Another COVID-19 nasopharyngeal swab was taken again which returned back as positive. As a result, the patient was treated symptomatically with paracetamol, vitamin C tablets, and zinc supplements, and was advised for home isolation with daily symptoms follow-up by the hospital infection control team (Table 3).

**Table 3:** Sequence of the second case test results.

Dates	19/04/2020	09/05/2020	12/07/2020
COVID-19 test result	Positive	Negative	Positive

**Figure 3:**
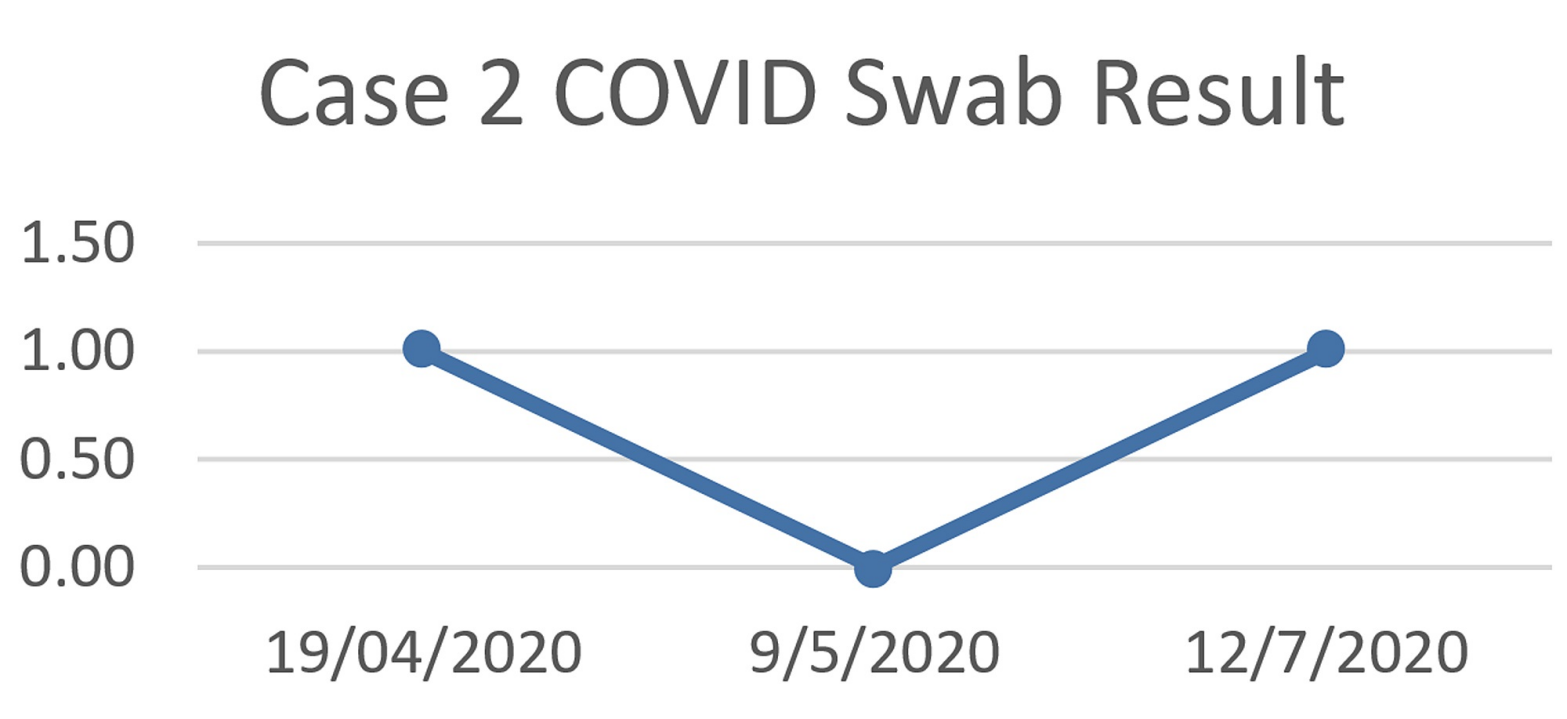
Sequence of the first case COVID-19 test results.

During our team follow up in home isolation his symptoms subsided gradually within a five days duration. Particularly. the fever and cough had resolved on the second day of home isolation, while other symptoms including loss of taste and body ache continued for a total of five days (Figure 3).

## Discussion

Reinfection, relapse, recurrence, and reactivation are terms that have been the source of conflict among cases which had a positive swab after showing resolution of symptoms with a negative swab results [4]. Gousseff et al. reported 11 patients who had a longer median time of symptomatic COVID-19 course ranging between mild and moderate severity, who all were healthcare workers with suspicion of recurrence [6]. Furthermore, in South Korea 116 reported cases where the COVID-19 patients after confirmed negative swabs and resolution of symptoms showed reactivation of the virus weeks later. The South Korean Centers for Disease Control and Prevention attributed these to reactivation rather than reinfection of the disease as many reported cases globally were reporting long persistent course of disease even after resolution of the symptoms [4, 7-8]. Similarly, Song et al. reported a case of a 30-year-old male patient who had seven consecutive false negative tests of COVID-19 while he had clear symptoms of COVID-19 such as fever, shortness of breath, and cough [9]. As a result, the hypothesis of which reinfection is possible is yet to be proven as early stages and mild courses of the disease translate to the low activity of the virus within the body. In our two cases, we hypothesize the negative swab COVID-19 result as a case of low quantitative viral assays or viral activity as both of them presented at early stages with only one or two symptoms, in which its severity increased with time moderately but was resolved with conservative treatments.

## Conclusions

The reinfection status of COVID-19 is yet to be proven. We attribute the reason of low consecutive COVID-19 swabs to the early stages of the viral disease in young adults as their immune system plays a crucial role in suppressing the virus. However, if the viral activity increased in severity, the COVID-19 testing will be positive.

## References

[1] Arafkas M, Khosrawipour T, Kocbach P (2020). Current meta-analysis does not support the possibility of COVID-19 reinfections. J Med Virol.

[2] Koyama T, Platt D, Parida L (2020). Variant analysis of SARS-CoV-2 genomes. Bull WHO.

[3] Ota M (2020). Will we see protection or reinfection in COVID-19?. Nat Rev Immunol.

[4] Alizargar J (2020). Risk of reactivation or reinfection of novel coronavirus (COVID-19). J Formos Med Assoc.

[5] Carfì A, Bernabei R, Landi F (2020). Persistent symptoms in patients after acute COVID-19. JAMA.

[6] Gousseff M, Penot P, Gallay L (2020). Clinical recurrences of COVID-19 symptoms after recovery: Viral relapse, reinfection or inflammatory rebound?. J Infect.

[7] D'Ardes D, Boccatonda A, Rossi I (2020). Long-term positivity to SARS-CoV- 2: a clinical case of COVID-19 with persistent evidence of infection. Eur J Case Rep Intern Med.

[8] Luo A (2020). Positive SARS-Cov-2 test in a woman with COVID-19 at 22 days after hospital discharge: a case report. J Traditional Chin Med Sci.

[9] Song C-Y, Yang D-G, Lu Y-Q (2020). A COVID-19 patient with seven consecutive false-negative rRT-PCR results from sputum specimens. Intern Emerg Med.

